# Author Correction: Ybx1 fine-tunes PRC2 activities to control embryonic brain development

**DOI:** 10.1038/s41467-023-36069-z

**Published:** 2023-01-25

**Authors:** Myron K. Evans, Yurika Matsui, Beisi Xu, Catherine Willis, Jennifer Loome, Luis Milburn, Yiping Fan, Vishwajeeth Pagala, Jamy C. Peng

**Affiliations:** 1 Department of Developmental Neurobiology, St. Jude Children’s Research Hospital, 262 Danny Thomas Place, Memphis, TN 38105 USA; 2 Center for Applied Bioinformatics, St. Jude Children’s Research Hospital, 262 Danny Thomas Place, Memphis, TN 38105 USA; 3 Center for Proteomics and Metabolomics, St. Jude Children’s Research Hospital, 262 Danny Thomas Place, Memphis, TN 38105 USA

**Keywords:** Methylation, Embryogenesis, Developmental neurogenesis, Histone post-translational modifications, Epigenetics in the nervous system

Correction to: *Nature Communications* 10.1038/s41467-020-17878-y, published online 13 August 2020

The original version of this Article contained an error in Fig. 6d, in which an incorrect western blot was included. This has been corrected in both the PDF and HTML versions of the Article.

