# Supplementary material for: Ybx1 fine-tunes PRC2 activities to control embryonic brain development
Source: Nat Commun. 2020 Aug 13;11:4060. doi: 10.1038/s41467-020-17878-y (PMC7426271; doi:10.1038/s41467-020-17878-y)
Supplement: Supplementary file 1 — Supplementary Information [file 41467_2020_17878_MOESM1_ESM.pdf]

Supplementary Information

Ybx1 fine-tunes PRC2 activities to control embryonic brain development

Evans *et al.*

Contents

Supplementary Figure 1-9
Supplementary Table 1-4

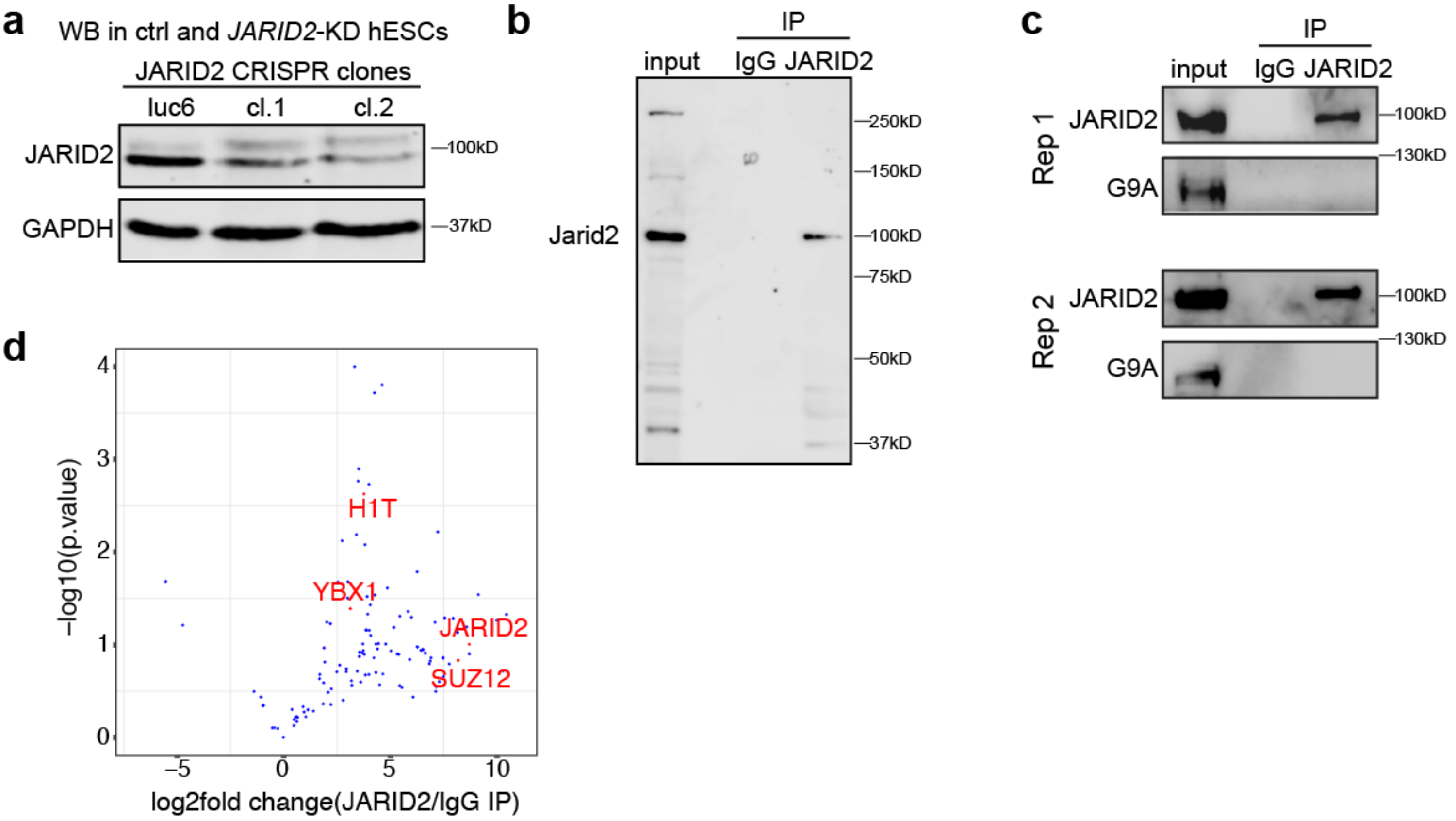

e

Proteins	peptide #, **IgG** IP1	IP2	IP3	spectral count, **IgG** IP1	IP2	IP3	peptide #, **JARID2** IP1	IP2	IP3	spectral count, **JARID2** IP1	IP2	IP3	p value IP1	IP2	IP3
JARID2	0	0	0	0	0	0	14	27	15	47	63	75	1e-15	2e-20	9e-6
SUZ12	0	0	0	0	0	0	6	24	11	23	53	55	2e-8	2e-17	2e-4
EZH2	0	0	0	0	0	0	6	16	0	13	22	0	3e-5	6e-8	n.a.
EED	0	0	0	0	0	0	5	6	0	13	19	0	4e-5	5e-7	n.a.
AEBP2	0	0	0	0	0	0	9	3	0	6	15	0	6e-3	9e-6	n.a.
RBBP4	0	0	0	0	0	0	6	8	2	13	12	10	3e-5	8e-5	1e-1
YBX1	0	0	0	0	0	0	7	4	15	35	15	75	6e-6	8e-6	9e-6

Supplementary Figure 1 – Validation of antibodies and Jarid2 IP-mass spectrometry.
WB analysis of (a) control and JARID2-knockdown (KD) hESCs and (b, c) IgG and JARID2 co-IP from the hESC nuclear extract. (d) Volcano plot of triplicate IgG and Jarid2 IP-mass spectrometry from the hESC nuclear extract. *P* values were calculated by 2-tailed t test comparing the abundance values of JARID2 vs. IgG IP. Abundance was calculated with the formula of spectral count × 50kD / protein size kD. (e) Mass spectrometry peptide counts and spectral counts of proteins identified in replicate JARID2 and IgG co-immunoprecipitates from the hESC nuclear extract. Spectral count represents the total number of MS/MS spectra that were matched to an assigned protein. *P* values were calculated by 2-tailed G test (Zhou et al. J Proteome Res. 2010, 9:5133). Source data are provided in Source Data file.

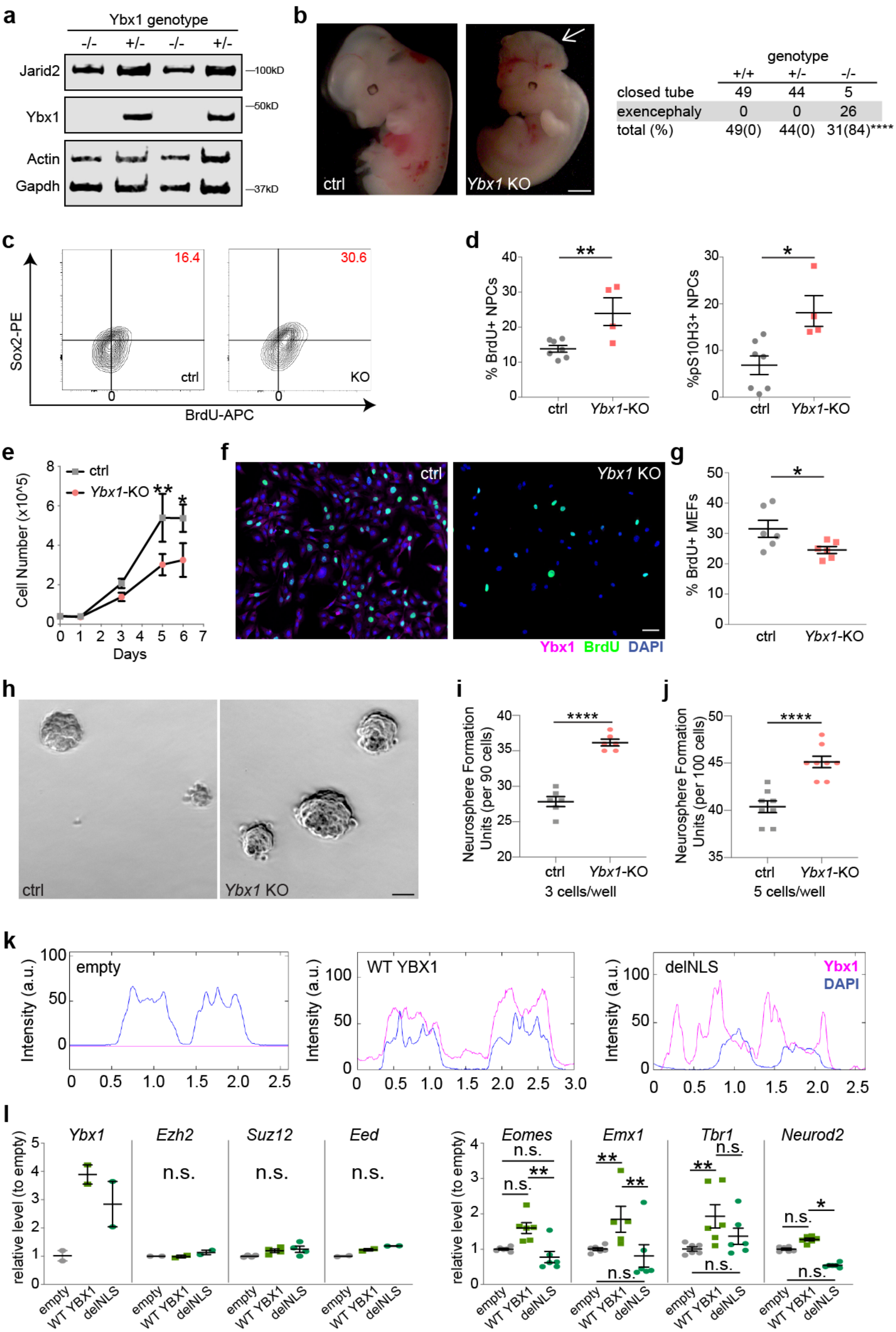
a
Ybx1 genotype
-/- +/- -/- +/-
Jarid2
Ybx1
Actin
Gapdh
100kD
50kD
37kD
b
ctrl
Ybx1 KO
genotype
+/+ +/- -/-
closed tube 49 44 5
exencephaly 0 0 26
total (%) 49(0) 44(0) 31(84)****
c
16.4
30.6
Sox2-PE
BrdU-APC
ctrl
KO
d
% BrdU+ NPCs
%pS10H3+ NPCs
ctrl
Ybx1-KO
e
ctrl
Ybx1-KO
Cell Number (x10^5)
Days
f
ctrl
Ybx1 KO
Ybx1 BrdU DAPI
g
% BrdU+ MEFs
ctrl
Ybx1-KO
h
ctrl
Ybx1 KO
i
Neurosphere Formation Units (per 90 cells)
ctrl
Ybx1-KO
3 cells/well
j
Neurosphere Formation Units (per 100 cells)
ctrl
Ybx1-KO
5 cells/well
k
empty
WT YBX1
delNLS
Ybx1
DAPI
Intensity (a.u.)
l
Ybx1
Ezh2
Suz12
Eed
Eomes
Emx1
Tbr1
Neurod2
relative level (to empty)
n.s.
empty
WT YBX1
delNLS

Supplementary Figure 2 – *Ybx1*-KO neural tubes and fibroblasts had different proliferation properties. (a) WB analysis of mNPC nuclear extract from *Ybx1*-KO or sibling control embryos. (b) Representative images of *Ybx1*-KO and sibling control embryos at E13.5. Arrow indicates exencephaly. Quantification of exencephaly in wild-type, heterozygous *Ybx1*-KO/+, and *Ybx1*-KO embryos. Numbers in parentheses indicate % of exencephaly. **** indicates *P* <0.0001 by 2-sided chi-square test. (c) Representative FACS analysis of Sox2 (y-axis) and BrdU (x-axis) to identify proliferative NPCs from *Ybx1*-KO and sibling control embryos. Quantification of FACS of (d) BrdU- or PH3-positive NPCs from *Ybx1*-KO and sibling control embryos (n=7 control and *Ybx1*-KO). (e) Quantification of the numbers of mouse embryonic fibroblasts (MEFs) *fromYbx1*-KO and sibling control (n=3). * indicates *P* < 0.05 by one-sided Student's *t* test. (f) IF of Ybx1 and BrdU in *Ybx1*-KO and sibling control MEFs. Bar, 50 µm. (g) Quantification of BrdU-positive MEFs from *Ybx1*-KO and sibling control (n=6 images examined over 3 biological replicates). * indicates *P* < 0.05 by one-sided Student's *t* test. (h) Representative images of neurospheres formed by *Ybx1*-KO and sibling control NPCs. Bar, 200 µm. Quantification of clonal neurosphere assay plated at (i) 3 or (j) 5 cells per well. **** indicates *p*<0.0001 by 2-tailed unpaired *t* test. (k) Line profiles of Ybx1 IF signals with DAPI signals from zoom-in cells in Fig. 1j. Profiles are normalized to maximum fluorescence signals. (l) RT-qPCR analysis of gene expression in *Ybx1*-KO NPCs transduced with empty control, wild-type YBX1, or delNLS-YBX1 (left, n=2/indicated line; right, n=6/indicated line). n.s. indicates not significant. *, **, ***, and **** indicate *P* <0.05, 0.01, 0.001, and 0.0001. Data are presented as mean +/- SEM for d, e, g, j, k. *p*-values by two-tailed unpaired *t* test are indicated in d and g, by two-way ANOVA (repeated measurements) followed by Bonferroni's multiple comparison post hoc test in e and by ordinary two-way ANOVA followed by Holm-Sidak multiple comparison test in k. Source data are provided in Source Data file. *P*-values: 2d(BrdU)-0.008 ,2d(pS10H3)-0.01 ;2e(Day 5)-0.009, 2e(Day6)-0.02; 2g-0.04; 2i-<0.0001; 2j-<0.0001; 2l(Eomes WT-delNLS)-0.009, (Emx1 empty-WT)-0.007, (Emx1 WT-delNLS)-0.001, (Tbr1 empty-WT)-0.003, (NeuroD2 WT-delNLS)-0.02.

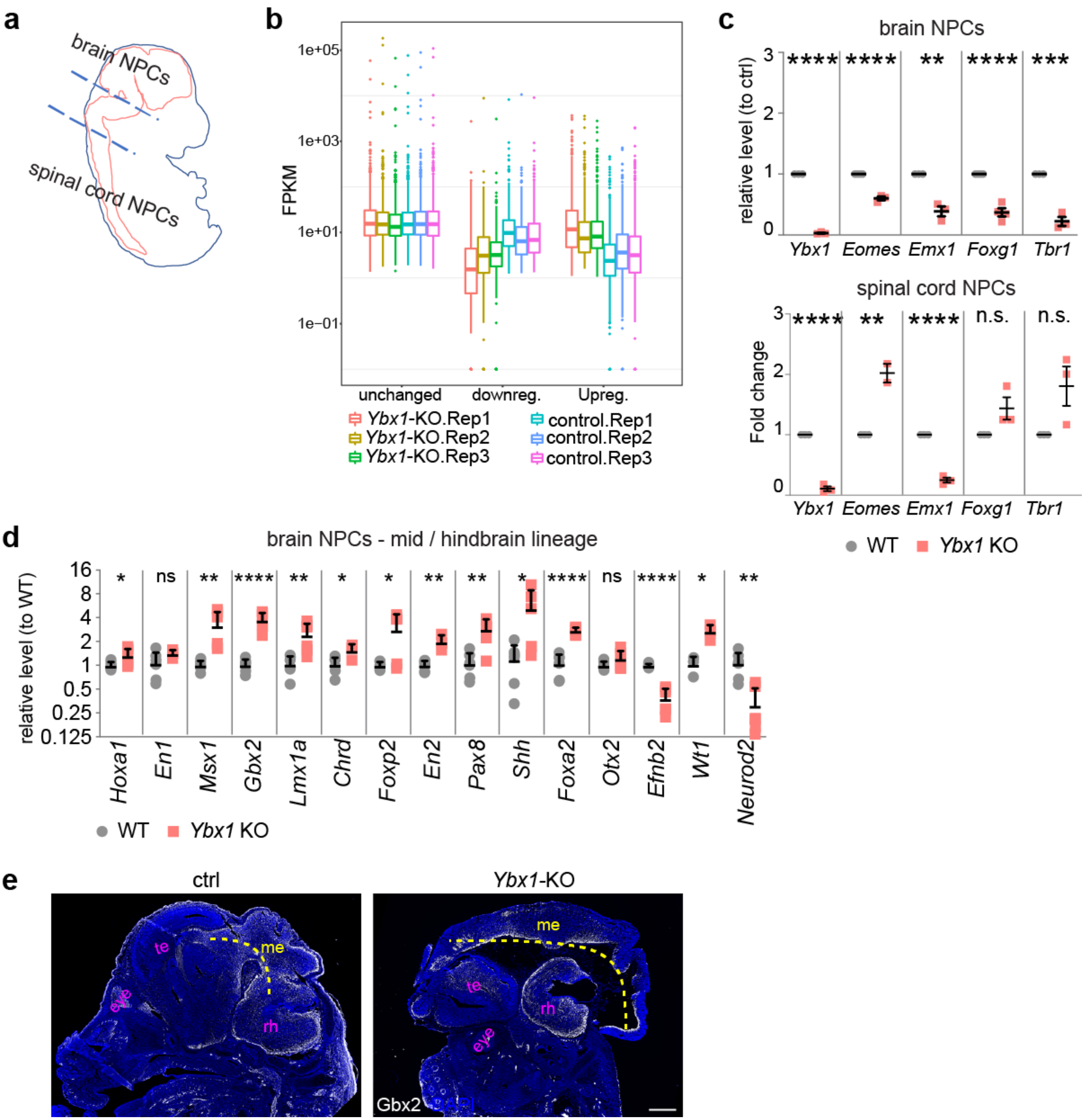

Supplementary Figure 3 – Analyses of *Ybx1*-KO and control NPCs and embryos.
(a) Schematic diagram of dissection to isolate brain and spinal cords. (b) Boxplot of unchanged, downregulated, and upregulated genes in control and *Ybx1*-KO NPC RNA-seq datasets (n=3). Minima, centre, and maxima of box indicate 25-, 50-, and 75- percentile, respectively. (c) RT-qPCR analysis of *Ybx1* and forebrain lineage markers in NPCs purified from brains or spinal cords of *Ybx1*-KO or sibling control embryos at E13.5 (n=3 for brain and SC [*Foxg1*-n=4]). (d) RT-qPCR with TaqMan assays of midbrain and hindbrain lineage markers in NPCs purified from brains of *Ybx1*-KO or sibling control embryos at E13.5 (n=6). (e) Gbx2 IF in cryosection from *Ybx1*-KO or sibling control embryos at E13.5. Bar, 500 µm. Te, telencephalon; me,

mesencephalon; np, nasal plate; and rh, rhombencephalon. Error bars are standard errors. n.s. indicates not significant. *, **, ***, and **** indicate P <0.05, 0.01, 0.001, and 0.0001. Source data are provided in Source Data file. Mean +/- SEM for c and d. p-values by two-tailed unpaired t test are indicated in c and d. P-values: 3c(brain Ybx1)-<0.0001, (brain Eomes)-<0.0001, (brain Emx1)-0.002, (brain Foxg1)-<0.0001, (brain Tbr1)-0.0005, (SC Ybx1)-<0.0001, (SC Eomes)-0.003, (SC Emx1)-<0.0001; 3d(Hoxa1)-0.04, (Msx1)-0.008, (Gbx2)-<0.0001, (Lmx1a)-0.008, (Chrd)-0.03, (Foxp2)-0.03, (En2)-0.002, (Pax8)-0.003, (Shh)-0.03, (Foxa2)-<0.0001, (Efnb2)-<0.0001, (Wt1)-0.01, (NeuroD2)-0.002.

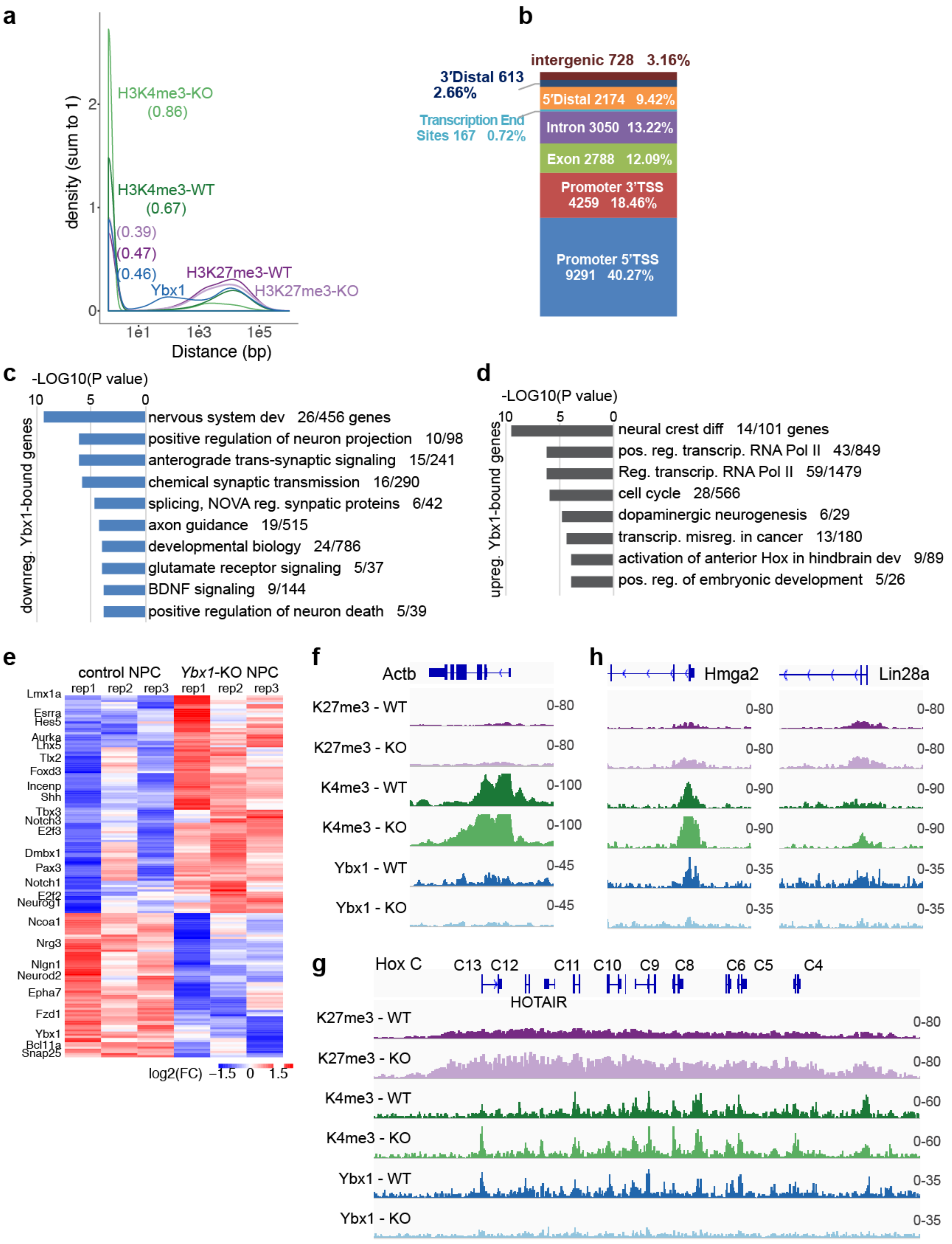

a
density (sum to 1)
H3K4me3-KO
(0.86)
H3K4me3-WT
(0.67)
(0.39)
(0.47)
(0.46)
H3K27me3-WT
Ybx1
H3K27me3-KO
Distance (bp)
b
intergenic 728 3.16%
3'Distal 613
2.66%
5'Distal 2174 9.42%
Transcription End Sites 167 0.72%
Intron 3050 13.22%
Exon 2788 12.09%
Promoter 3'TSS 4259 18.46%
Promoter 5'TSS 9291 40.27%
c
-LOG10(P value)
downreg. Ybx1-bound genes
nervous system dev 26/456 genes
positive regulation of neuron projection 10/98
anterograde trans-synaptic signaling 15/241
chemical synaptic transmission 16/290
splicing, NOVA reg. synpatic proteins 6/42
axon guidance 19/515
developmental biology 24/786
glutamate receptor signaling 5/37
BDNF signaling 9/144
positive regulation of neuron death 5/39
d
-LOG10(P value)
upreg. Ybx1-bound genes
neural crest diff 14/101 genes
pos. reg. transcrip. RNA Pol II 43/849
Reg. transcrip. RNA Pol II 59/1479
cell cycle 28/566
dopaminergic neurogenesis 6/29
transcrip. misreg. in cancer 13/180
activation of anterior Hox in hindbrain dev 9/89
pos. reg. of embryonic development 5/26
e
control NPC
Ybx1-KO NPC
rep1 rep2 rep3 rep1 rep2 rep3
Lmx1a
Esrra
Hes5
Aurka
Lhx5
Tlx2
Foxd3
Incenp
Shh
Tbx3
Notch3
E2f3
Dmbx1
Pax3
Notch1
E2f2
Neurog1
Ncoa1
Nrg3
Nlgn1
Neurod2
Epha7
Fzd1
Ybx1
Bcl11a
Snap25
log2(FC) −1.5 0 1.5
f
Actb
K27me3 - WT
K27me3 - KO
K4me3 - WT
K4me3 - KO
Ybx1 - WT
Ybx1 - KO
0-80
0-80
0-100
0-100
0-45
0-45
h
Hmga2
Lin28a
0-80
0-80
0-90
0-90
0-35
0-35
g
Hox C
C13 C12 C11 C10 C9 C8 C6 C5 C4
HOTAIR
K27me3 - WT
K27me3 - KO
K4me3 - WT
K4me3 - KO
Ybx1 - WT
Ybx1 - KO
0-80
0-80
0-60
0-60
0-35
0-35

Supplementary Figure 4 – Histone ChIP-seq and Ybx1 CUT&RUN-seq

(a) Kernel density plots of histone ChIP-seq peaks or Ybx1 CUT&RUN-seq peaks relative to transcription start sites (0-bp position). Area under the curve values sum to 1, with total peaks normalized to 1. Numbers in parentheses indicate areas under the curves that are within 10 bp of transcription start sites. (b) Box plot indicates the proportions of Ybx1 binding site enrichment. “Distal” indicates regions that are 2–50kb from gene bodies. “Tes” denotes transcription termination sites. Gene ontology analysis of (c) downregulated or (d) upregulated Ybx1 target genes in *Ybx1*-KO NPCs. Ontology terms were ranked by *P* values, which were calculated by 2-tailed Fisher’s exact test, with the number of bound genes indicated. (e) Unsupervised clustering of Ybx1-bound genes that became differentially expressed in control vs. *Ybx1*-KO NPCs. H3K27me3 and H3K4me3 ChIP-seq and Ybx1 CUT&RUN-seq tracks at (f) *Actb*, (g) *Hox* C cluster, and (h) upregulated genes *Hmga2* and *Lin28a* in control and *Ybx1*-KO NPCs.

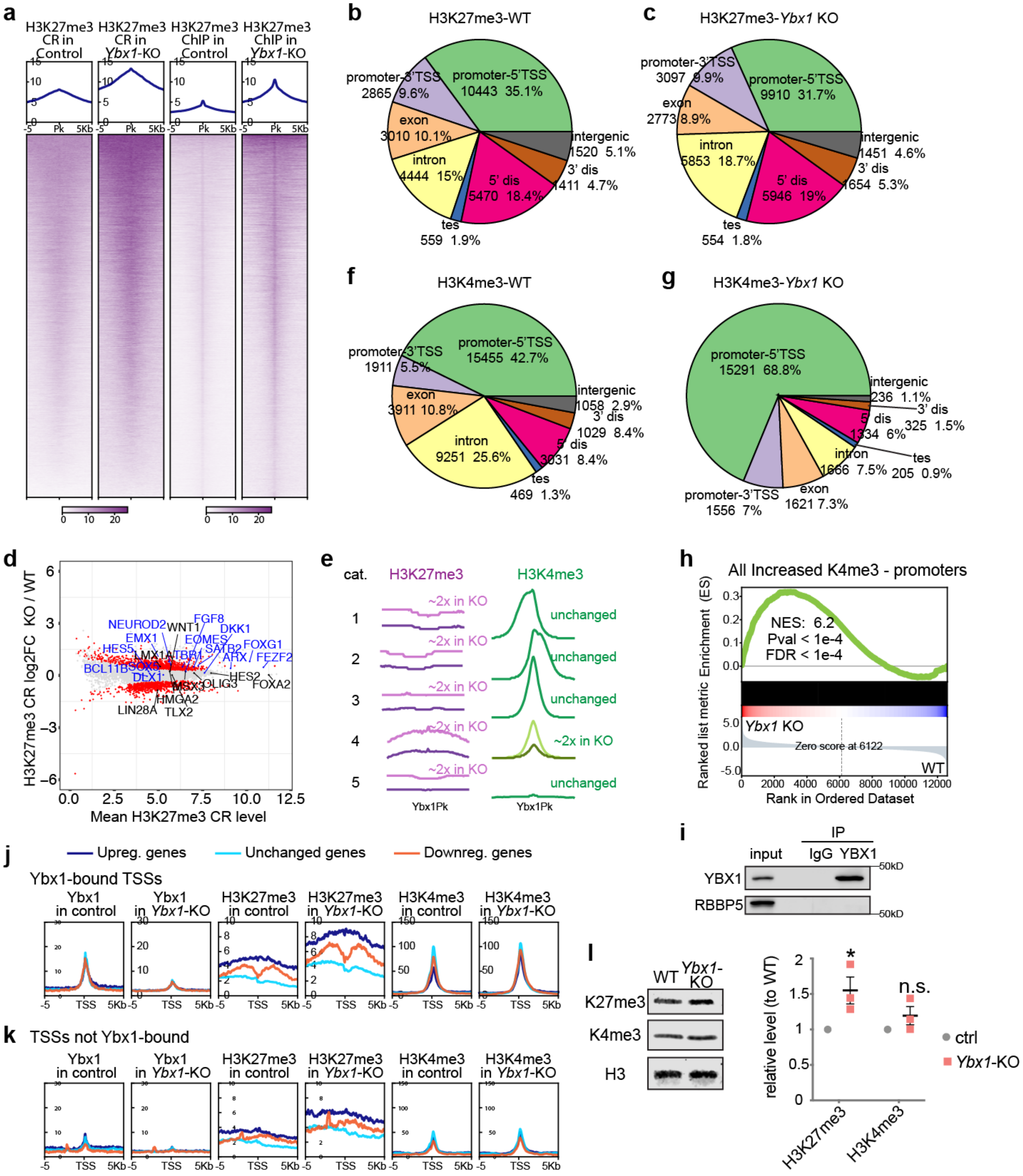

Supplementary Figure 5 – H3K27me3 and H3K4me3 profiling in control and *Ybx1*-KO NPCs.
(a) Heat maps of H3K27me3 CUT&RUN-seq and ChIP-seq in control and *Ybx1*-KO NPCs. Pie charts indicate the proportions of (b, c) H3K27me3-occupied or (f, g) H3K4me3-occupied site enrichment in WT and *Ybx1*-KO

NPCs. (d) MA plot of CUT&RUN-seq data: LOG2(H3K27me3 fold-changes of *Ybx1*-KO / WT NPCs) vs. mean H3K27me3 levels at individual genes. Red dots are significant changes passing the FDR-adjusted $p<0.05$ and fold change>2. Blue indicates downregulated genes and black indicates upregulated genes in *Ybx1*-KO NPCs. *P* values were calculated by 2-tailed empirical Bayes moderated t-statistic after Trimmed Mean of M-values normalization. (e) Graphical description of the 5 categories of Ybx1-bound regions that were defined by H3K27me3 and H3K4me3 distribution in control and *Ybx1*-KO NPCs. (h) GSEA of genes with significantly increased levels of H3K4me3 at promoters. *P* values were calculated by 1-tailed Kolmogorov-Smirnov statistic test. (i) WB analysis of IgG and Ybx1 co-IP from hESC nuclear extract. Average profiles of Ybx1, H3K27me3, and H3K4me3 distribution at (j) Ybx1-bound TSSs or (k) other TSSs of upregulated, unchanged, or downregulated genes in control and *Ybx1*-KO NPCs. (l) WB analysis of histones and quantification in WT and *Ybx1*-KO neural tube cells. n=3. Data presented as mean +/- SEM. *p*-value by two-tailed unpaired *t* test. * indicates $P<0.05$ by the chi-square test. Source data are provided in Source Data file. *P*-value 5l: 0.04

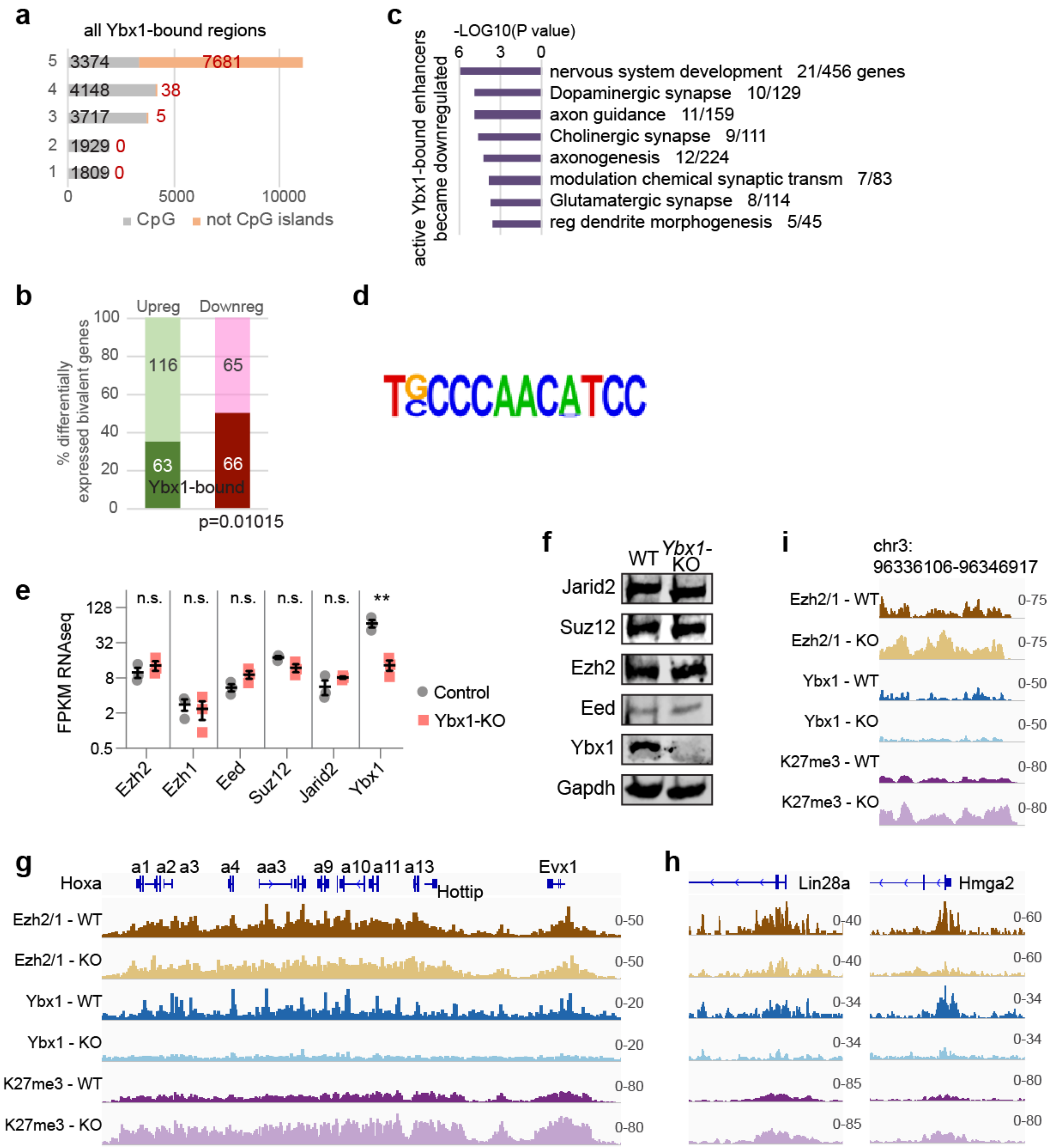

Supplementary Figure 6 – Ybx1-bound genes and Ezh2/1 CUT&RUN-seq in control and *Ybx1*-KO NPCs. (a) Proportions of Ybx1-bound regions that overlap CpG islands. (b) Proportions of differentially expressed genes in *Ybx1*-KO that were Ybx1-bound in control NPCs. *p*-value by Fisher's exact test (c) Gene ontology analysis of Ybx1-bound active enhancers (co-occupied by H3K27ac and H3K4me1) that became downregulated in *Ybx1*-KO NPCs. Ontology terms were ranked by *P* values, which were calculated by 2-tailed Fisher's exact test, with the number of bound genes indicated. (d) Ybx1 DNA binding motif were enriched in

upregulated genes in *Ybx1*-KO with *p*<1e-6 calculated by 2-tailed hypergeometric test. (e) FPKM values of PRC2 subunits and Ybx1 in control and *Ybx1*-KO RNA-seq datasets. n=3 Data presented as mean +/- SEM. *p*-values by two-tailed unpaired *t* test. Data presented as mean +/- SEM. *p*-values by two-tailed unpaired *t* test. (f) WB analysis of control and *Ybx1*-KO NPCs. Ezh2/1 and Ybx1 CUT&RUN-seq tracks and H3K27me3 ChIP-seq tracks at (g) *Hox* A cluster, (h) *Lin28a* and *Hmga2* loci, and (i) an ectopic Ezh2/1-bound region in control and *Ybx1*-KO NPCs. n.s. and * indicate not significant and *p*<0.05. Source data are provided in Source Data file. *P*-values: 6e(Ybx1)-0.006.

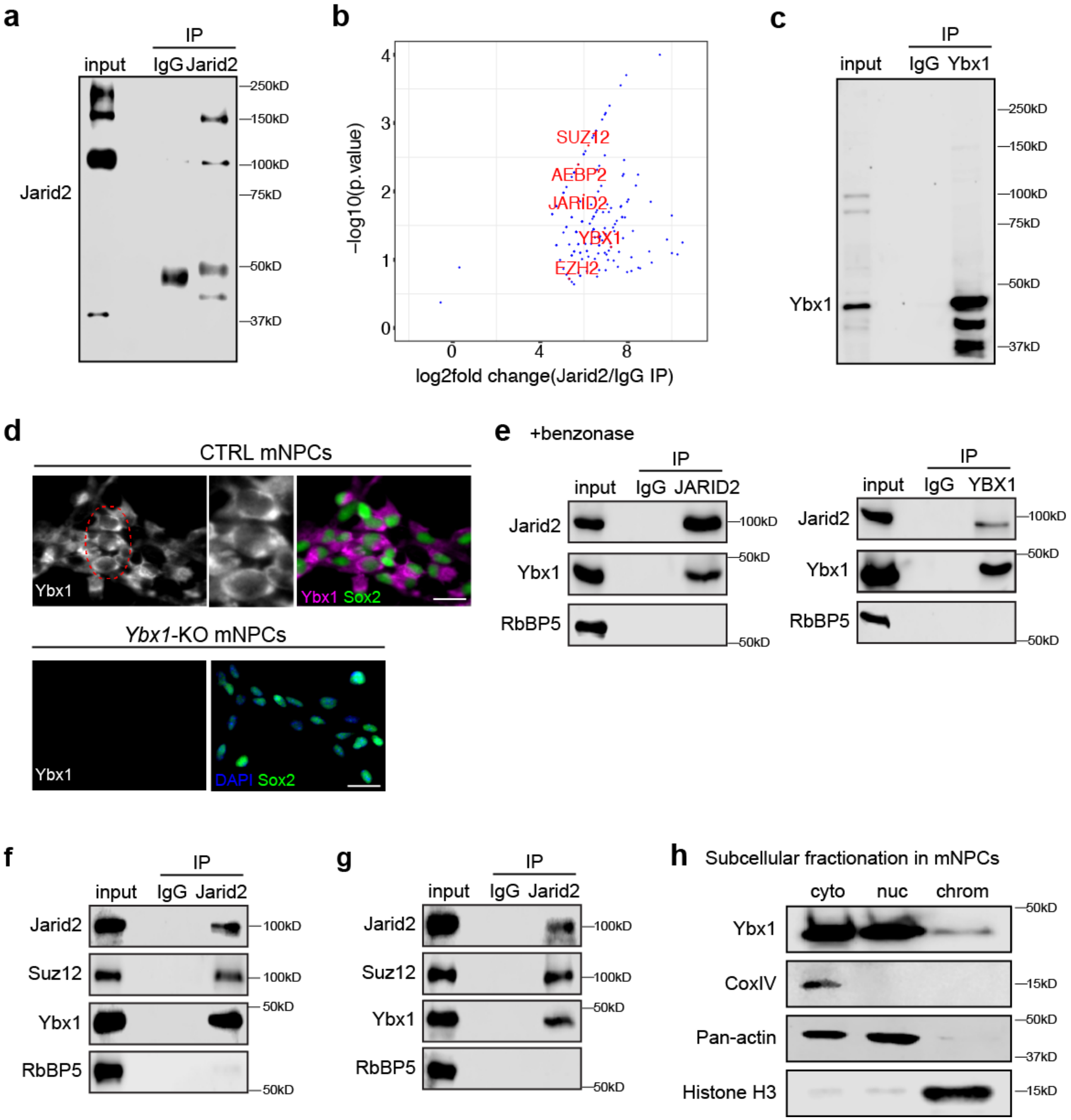

Supplementary Figure 7 – Validation of antibodies, IP-mass spectrometry, and coIP-WB.
(a) WB and (b) mass spectrometry analysis of triplicate IP using IgG and a second JARID2 (R&D Systems AF6090) antibody from the mouse NE4C nuclear extract. P values were calculated by 2-tailed t test comparing the abundance values of Jarid2 vs. IgG IP. (c) WB analysis of IgG and Ybx1 co-IP in the hESC nuclear extract. (d) IF of Ybx1 and Sox2 in NPCs isolated from *Ybx1*-KO or sibling control embryos at E13. Bar, 50 or 25 µm. WB analysis of co-IP in the hESC nuclear extract using (e) benzonase treatment, (f) the second JARID2 (R&D Systems AF6090) antibody, and (g) the third JARID2 (EMD Millipore ABE425) antibody. (h) WB analysis of subcellular fractionation of NPCs. Source data are provided in Source Data file.

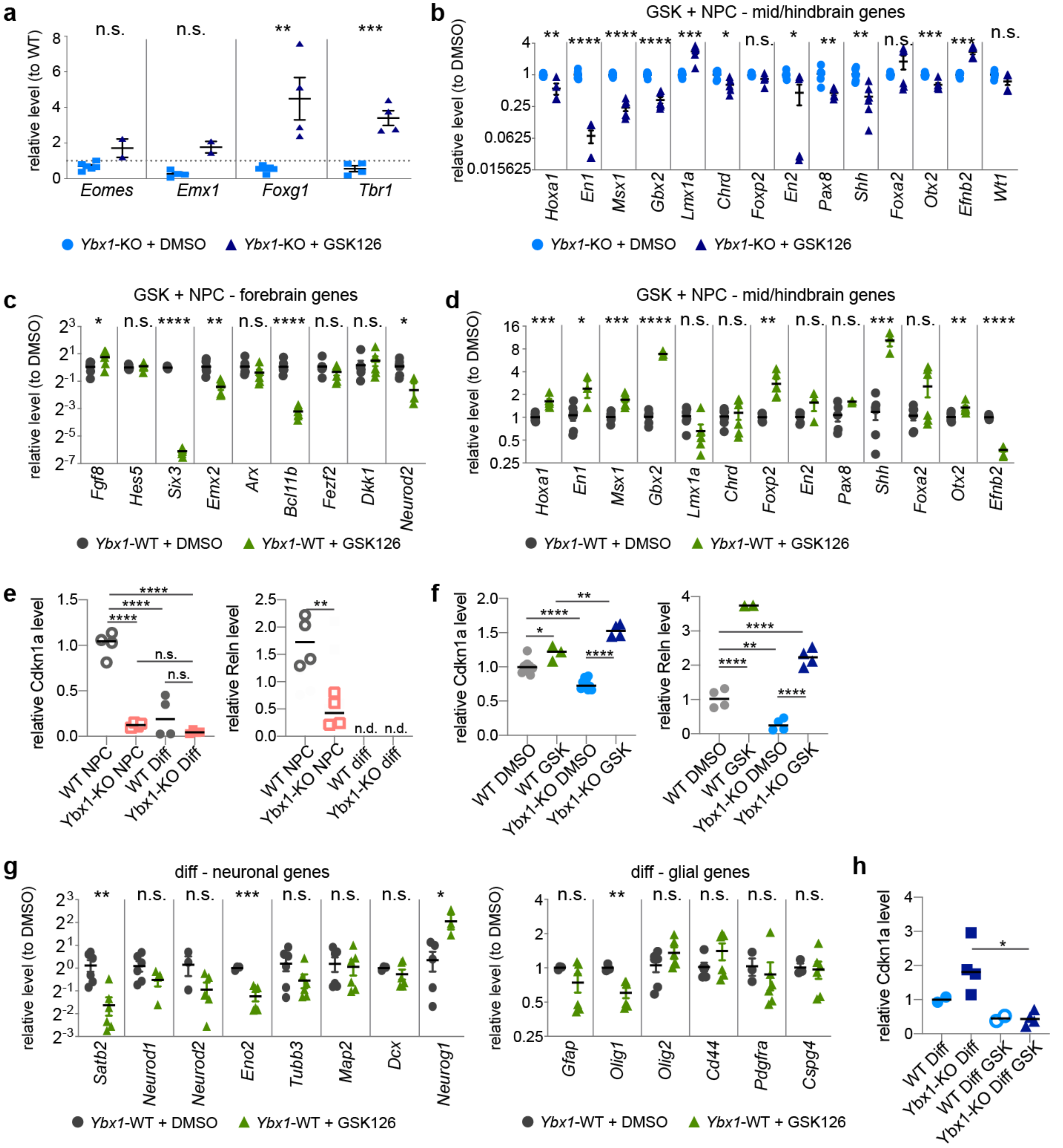

Supplementary Figure 8 – Analyses of GSK126 treatment of control and *Ybx1*-KO NPCs.
Ybx1-KO NPCs were treated for 4 days and their transcripts analyzed by RT-qPCR with (a, n=6 DMSO, n=4 GSK126) primers or (b, n=6/treatmen) TaqMan assays. (c, d) Control NPCs were treated for 4 days and their transcripts analyzed by RT-qPCR with TaqMan assays (n=6/treatment [*En1*, *En2*, *Pax8*, *Shh*-n=3/GSK126]). (e) Control and *Ybx1*-KO NPCs and neural differentiation were analyzed for Cdkn1a and Reln expression (n=4/treatment). RT-qPCR analysis of control and *Ybx1*-KO NPCs treated with DMSO or 500µM GSK126 (f) after 4 days (*Cdkn1a* n=10/DMSO, 3/GSK126(ctrl), 4/GSK126(*Ybx1*-KO); *Reln*=n=4/DMSO, 2/GSK126(ctrl),

4/GSK126(*Ybx1*-KO)) (h) and after differentiation (n=2 (ctrl) and 4 (*Ybx1*-KO)). (g) RT-qPCR with TaqMan assays of *Ybx1*-KO differentiating cells treated with DMSO or 500μM GSK126 after 4 days. (n=6/treatment [*see source data*]). Data are presented as mean +/- SEM for b-h. *p*-values by two-tailed unpaired *t* test are indicated in b, c, d, and g and by ordinary one-way ANOVA followed by Tukey's post hoc test in e, f, and h. n.s. indicates not significant. *, **, ***, and **** indicate $P < 0.05$, 0.01, 0.001, and 0.0001, respectively. Source data are provided in Source Data file. *P*-values: 8a(Foxg1)-0.003, (Tbr1)-0.0007; 8b(Hoxa1)-0.004, (En1)-<0.0001, (Msx1)-<0.0001, (Gbx2)-<0.0001, (Lmx1a)-0.0003, (Chrd)-0.01, (En2)-0.02, (Pax8)-0.004, (Shh)-0.002, (Otx2)-0.0003, (Efnb2)-0.0001; 8c(Fgf8)-0.03, (Six3)-<0.0001, (Emx2)-0.002, (Bcl11b)-<0.0001, (NeuroD2)-0.02; 8d(Hoxa1)-0.0006, (En1)-0.02, (Msx1)-0.0008, (Gbx2)-<0.0001, (Foxp2)-0.002, (Shh)-0.0001, (Otx2)-0.007, (Efnb2)-<0.0001; 8e(Cdkn1a WT NPC-KO NPC)-<0.0001, (Cdkn1a WT NPC-WT Diff)-<0.0001, (Cdkn1a WT NPC-KO Diff)-<0.0001, (Reln WT NPC-KO NPC)-0.003; 8f(Cdkn1a WT DMSO-GSK)-0.01, (Cdkn1a WT DMSO-KO DMSO)-<0.0001, (Cdkn1a WT GSK-KO GSK)-0.0004, (Cdkn1a KO DMSO-KO GSK)-<0.0001, (Reln WT DMSO-GSK)-<0.0001, (Reln WT DMSO-KO DMSO)-0.004, (Reln WT DMSO-KO GSK)-<0.0001, (Reln KO DMSO-GSK)-<0.0001; 8g(Satb2)-0.004, (Eno2)-0.0004, (NeuroG1)-0.01, (Olig1)-0.005; 8h (KO Diff-KO Diff GSK)-0.01.

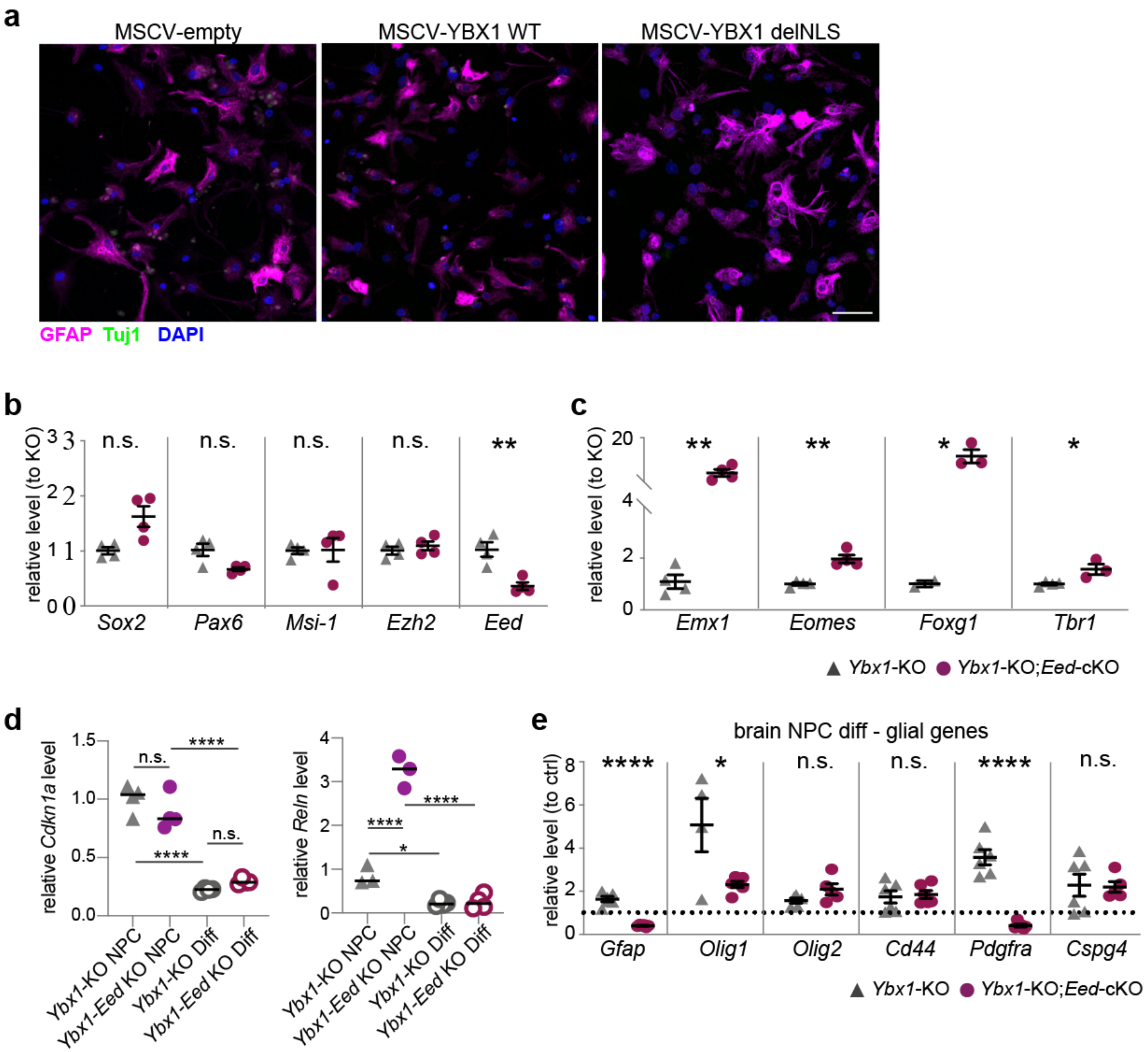

Supplementary Figure 9 – Analyses of *Ybx1*-KO and Ybx1-Eed-dKO NPCs and differentiating cells. (a) IF of the differentiation of *Ybx1*-KO NPCs transduced with lentiviruses to express empty negative control, wild-type *YBX1*, or delNLS-*YBX1*. Bar, 100 µm. (b) *Ybx1*-KO and *Ybx1-Eed*-dKO NPCs were analyzed by RT-qPCR using primers. NPC markers Sox2, Pax6, and Msi-1 did not differ between the groups. Ezh2 did not differ. Ybx1 levels were markedly downregulated in *Ybx1*-KO and Ybx1-Eed-dKO when compared with control. Eed in Ybx1-Eed-dKO was significantly downregulated when compared with *Ybx1*-KO (n=4/genotype). (c) In *Ybx1*-KO and Ybx1-Eed-dKO differentiating cells, forebrain markers Emx1, Eomes, and Foxg1 and neuronal marker Tbr1 were significantly upregulated (n=4 [*Foxg1* n=2 ctrl]). (d) Quantification of Cdkn1a and Reln levels in NPCs and differentiating cells (n=3/*Ybx1* KO; *Eed* WT NPC\ n=4/others). (e) RT-qPCR with TaqMan assays of glial genes in cells after 14 days of differentiation (n=6). n.s. indicates not significant. *, ***, and **** indicate $P < 0.05$, 0.001, and 0.0001, respectively. Source data are provided in Source Data file. *P*-values: 9b (Eed)-0.004; 8c (Emx1)-0.003, (Eomes)-0.001, (Foxg1)-0.03, (Tbr1)-0.02; 9d (Cdkn1a KO NPC-KO Diff)-<0.0001, (Cdkn1a dKO NPC-dKO Diff)-<0.0001, (Reln KO NPC-dKO NPC)-<0.0001, (Reln KO NPC-KO Diff)-0.01, (Reln dKO NPC-dKO Diff)-<0.0001; 9e (Gfap)-<0.0001, (Olig1)-0.02, (Pdgfra)-<0.0001.

Supplementary Table 1. Antibodies used in this study.

Antibody	Species	Source	Catalogue Number	Dilution
Anti-JARID2	Rabbit	Novus Biologicals	NB100-2214	IP (4μg)
Anti-JARID2	Sheep	R&D Systems	AF6090	IP (4μg)
Anti-JARID2	Rabbit	EMD Millipore	ABE425	IP (4μg)
Anti-Ybx1	Rabbit	Bethyl Laboratories	A303-230A	IP (4μg)<br>WB (1:1000)<br>Cut-Run (0.4μg)
Anti-Ybx1	Rabbit	Abcam	ab12148	IP (4μg)<br>WB (1:1000)
Anti-Ybx1	Rabbit	Sigma	Y0396	IF (1:100)
Anti-Suz12	Rabbit	Cell Signaling Technology	3737	WB (1:1000)
Anti-Ezh2	Rabbit	Cell Signaling Technology	4905	WB (1:1000)
Anti-Ezh2 (clone AC22)	Mouse	Active Motif	39875	Cut-Run (0.4μg)
Anti-Eed	Mouse	ThermoFisher	MA5-16314	WB (1:1000)
Anti-Eed	Rabbit	Invitrogen	PA5-34420	WB (1:1000)
Anti-RbBP5	Rabbit	Bethyl Laboratories	A300-109A	WB (1:1000)
Anti-G9a	Rabbit	Cell Signaling Technology	AF1979	WB (1:1000)
Anti-SOX2	Goat	Santa Cruz	Sc-17319	IF (1:200)
Anti-GAPDH	Mouse	EMD Millipore	MAB374	WB (1:2000)
Anti-beta actin	Mouse	Sigma Aldrich	A1978	WB (1:2000)
Anti-COX IV	Rabbit	Cell Signaling Technology	4844	WB (1:1000)
Anti-histone H3	Rabbit	Active Motif	61475	WB (1:1000)
Anti-SOX2 PE (clone 14A6A34)	Mouse	BioLegend	656104	FACS (1:250)
Anti-BrdU	Mouse	BioLegend	364106	IF (1:100)
Anti-BrdU APC (clone Bu20a)	Mouse	BioLegend	339808	FACS (1:500)
Anti-Phospho-S10-Histone H3-Alexa 488 (D2C8)	Mouse	Cell Signaling Technology	3465	FACS (1:1000)
Anti-FoxG1 (EPR18987)	Rabbit	Abcam	Ab196868	IF (1:100)
Anti-Gbx2	Rabbit	ProteinTech	21639-1-AP	IF (1:100)
Anti-GFAP	Rabbit	Cell Signaling Technologies	12389S	IF (1:100)
Anti-GFAP	Mouse	MilliporeSigma	1G3893	IF (1:250)
Anti-beta Tubulin	Mouse	Sigma Aldrich	T8660	IF (1:200)
Anti-Histone H3K27me3	Rabbit	Active Motif	39155	ChIP (1μg)<br>WB (1:1000)

				Cut-Run (0.4μg)
Anti-Histone H3K4me3	Rabbit	Diagenode	C15410003	ChIP (1ug)<br>WB (1:1000)<br>Cut-Run (0.4ug)
Drosophila spike in control anti-H2Av	Rabbit	Active Motif	61686	ChIP (0.5ul) or Cut-Run (0.2ul)

Supplementary Table 2. TaqMan assays or primers used for RT-qPCR.

**Assay ID**	**Gene Symbol(s)**		qPCR: Ybx1 F: GGATGGCAATGAAGAGGACAAA
Hs99999901_s1	18s rRNA		qPCR: Ybx1 R: CGTCTGCGTCGGTAATTGAAGT
Mm00439359_m1	Hoxa1		qPCR: Ezh2 F: GTGACCACAGGATAGGCATCT
Mm00432802_m1	Gadd45a		qPCR: Ezh2 R: CAAGGGATTTCCATTTCTCG
Mm02619580_g1	Actb		qPCR: Eomes F: TACGGCCAGGGTTCTCCGCTCTAC
Mm00473947_m1	Lmx1a		qPCR: Eomes R:GGGCCGGTTGCACAGGTAGACGTG
Mm00545903_m1	Arx		qPCR: Emx1 F: CTAAGCGGGGTTTCACCATAGA
Mm00440330_m1	Msx1		qPCR: Emx1 R: GCACTGGGGTGAGGATAGTTG
Mm00550241_m1	Emx2		qPCR: Foxg1 F: AGCGACGACGTGTTCATCG
Mm00438709_m1	En1		qPCR: Foxg1 R: CCCGTTGTAACTCAAAGTGCTG
Mm00438203_m1	Chrd		qPCR: Tbr1 F: CAAGGGAGCATCAAACAACA
Mm00494578_m1	Gbx2		qPCR: Tbr1 R: GTCCTCTGTGCCATCCTCAT
Mm00438922_m1	Fgf8		qPCR: Wnt1 F: GAACCCTTTTGCCATCCTGA
Mm00439311_g1	Hes5		qPCR: Wnt1 R: CACCTTCAAGAGTTGACCTC
Mm01237639_m1	Six3		qPCR: Hmga2 F: AGACCCAGAGGAAGACCCAAAG
Mm00475030_m1	Foxp2		qPCR: Hmga2 R: TTCAGTCTCCTGAGCAGGCTTC
Mm00440623_m1	Pax8		qPCR: Gfap F: CAATGCTGGCTTCAAGGAGACACG
Mm01976556_s1	Foxa2		qPCR: Gfap R: TCAGTTCAGCTGCCAGCGCCT
Mm00446859_m1	Otx2		qPCR: Olig1 F: CTCGCCCAGGTGTTTTGTTG
Mm01320619_m1	Fezf2		qPCR: Olig1 R: TATAAGCCTGCGCTACGACG
Mm01337048_m1	Wt1		qPCR: Olig2 F: TGGAGAGATGCGTTCGTTCC
Mm00480516_m1	Bcl11b		qPCR: Olig2 R: GTGCTCTGCGTCTCGTCTAA
Mm00440465_g1	Neurod2		qPCR: Tubb3 F: TCTGGCGCCTTTGGACACCTATT
Mm00438422_m1	Dkk1		qPCR: Tubb3 R: TTCTCACACTCTTTCCGCACGACA
Mm00436528_m1	Shh		qPCR: Map2 F: TGTGCTGTGTGCTCCAAGTT
Mm00438710_m1	En2		qPCR: Map2 R: GCTGGTGGTATGTTCTGGCT
Mm01257777_m1	Nfib		qPCR: Sox2 F: ACAGCATGTCCTACTCGCAG

Mm00438670_m1	Efnb2		qPCR: Sox2 R: ATGCTGATCATGTCCCGGAG
Mm01253033_m1	Gfap		qPCR: Nestin F: GTCTCAGGACAGTGCTGAGCCTTC
Mm00497537_s1	Olig1		qPCR: Nestin R: TCCCCTGAGGACCAGGAGTCTC
Mm01210556_m1	Olig2		qPCR: Pax6 F: GCGGAGTTATGATACCTACACC
Mm00507331_m1	Satb2		qPCR: Pax6 R: GAAATGAGTCCTGTTGAAGTGG
Mm01946604_s1	Neurod1		qPCR: Msi1 F: GATGGCTCCCCCTCCAGGTT
Mm01277161_m1	Cd44		qPCR: Msi1 R: CATTGGTGAAGGCTGTGGCA
Mm00440701_m1	Pdgfra		qPCR: Notch2 F: CGGACCAGCCTGAGAACCT
Mm01185009_gH	Eno2		qPCR: Notch2 R: CCTCAAGAAGCTTCGCGAAT
Mm00507257_m1	Cspg4		qPCR: Pax3 F: GGGAACTGGAGGCATGTTTA
Mm00727586_s1	Tubb3		qPCR: Pax3 R: GTTTTCCGTCCCAGCAATTA
Mm00440466_s1	Neurog1		qPCR: actin F: TCCTCCTGAGCGCAAGTACTCT
Mm00485231_m1	Map2		qPCR: actin R: CGGACTCATCGTACTCCTGCTT
Mm00438400_m1	Dcx		
Mm02384867_s1	Foxd3		
Mm00500463_m1	Irx3		
Mm00809934_s1	Foxo6		
Mm01307193_g1	Apoe		
Mm01166201_m1	Dlx6		
Mm00580743_m1	Epha3		
Mm01187817_m1	Igfbp3		
Mm00436500_m1	Sema5a		
Mm01318470_m1	Dlg2		
Mm01192933_g1	Ctgf		
Mm01326464_m1	Neurod6		

Supplementary Table 3. Primers used for making DNA constructs.

YBX1-F: AAAAAGCAGGCTTCatgagcagcgaggccgagac
YBX1-R: AGAAAGCTGGGTGTTAttactcagccccgccctgct
YBX1 F1-R: AGAAAGCTGGGTGTTAaactggaacaccaccaggac
YBX1 F2-F: AAAAAGCAGGCTTCggagatggagagactgtggag
YBX1(XhoI)F1-R: taagcactcgagaactggaacaccaccaggac
YBX1(XhoI)F3-F: tgaccctcgaggtgaggcagaatatgtatcgggga
YBX1 R2-R: AGAAAGCTGGGTGTTAtccccgatacatattctgcctcac
YBX1delNLS(XhoI)-F: AAAAACTCGAGccacagtattccaaccct
YBX1delNLS(XHoI)-R: AAAAACTCGAGggcctggccttcgggag
YBX1(BamHI)-F: tgaccggatccatgagcagcgaggccgagac

Supplementary Table 4. Primers used for CUT&RUN-qPCR.

CUT&RUNqPCR: intergenic region F: TCTCTCACCGACCACTTTCC
CUT&RUNqPCR: intergenic region R: GTGGCTGACGGTGACATTTT
CUT&RUNqPCR: Sox2 F: TTCATGCAAAACCCTCTGGC
CUT&RUNqPCR: Sox2 R: AATAAATGGGTTTCCGGCGG
CUT&RUNqPCR: Foxg1 F: GAAGGCCTCCACAGAACG
CUT&RUNqPCR: Foxg1 R: CAGGCCGGTACTTACAGCTT
CUT&RUNqPCR: Eomes F: CTACTCCATGGACAGCCTGAG
CUT&RUNqPCR: Eomes R: GGTACTGGAAGAGCGAGCA
