# Supplementary material for: Ybx1 fine-tunes PRC2 activities to control embryonic brain development
Source: Nat Commun. 2020 Aug 13;11:4060. doi: 10.1038/s41467-020-17878-y (PMC7426271; doi:10.1038/s41467-020-17878-y)
Supplement: Supplementary file 3 — Reporting Summary [file 41467_2020_17878_MOESM3_ESM.pdf]

nature research

Corresponding author(s): Jamy C Peng, PhD

Last updated by author(s): 06/16/2020

# Reporting Summary

Nature Research wishes to improve the reproducibility of the work that we publish. This form provides structure for consistency and transparency in reporting. For further information on Nature Research policies, see Authors & Referees and the Editorial Policy Checklist .

## Statistics

For all statistical analyses, confirm that the following items are present in the figure legend, table legend, main text, or Methods section.

| n/a | Confirmed | |
|---|---|---|
| ☐ | ☒ | The exact sample size (*n*) for each experimental group/condition, given as a discrete number and unit of measurement |
| ☐ | ☒ | A statement on whether measurements were taken from distinct samples or whether the same sample was measured repeatedly |
| ☐ | ☒ | The statistical test(s) used AND whether they are one- or two-sided<br>*Only common tests should be described solely by name; describe more complex techniques in the Methods section.* |
| ☐ | ☒ | A description of all covariates tested |
| ☐ | ☒ | A description of any assumptions or corrections, such as tests of normality and adjustment for multiple comparisons |
| ☐ | ☒ | A full description of the statistical parameters including central tendency (e.g. means) or other basic estimates (e.g. regression coefficient) AND variation (e.g. standard deviation) or associated estimates of uncertainty (e.g. confidence intervals) |
| ☐ | ☒ | For null hypothesis testing, the test statistic (e.g. *F*, *t*, *r*) with confidence intervals, effect sizes, degrees of freedom and *P* value noted<br>*Give P values as exact values whenever suitable.* |
| ☒ | ☐ | For Bayesian analysis, information on the choice of priors and Markov chain Monte Carlo settings |
| ☐ | ☒ | For hierarchical and complex designs, identification of the appropriate level for tests and full reporting of outcomes |
| ☒ | ☐ | Estimates of effect sizes (e.g. Cohen's *d*, Pearson's *r*), indicating how they were calculated |

*Our web collection on statistics for biologists contains articles on many of the points above.*

## Software and code

Policy information about availability of computer code

Data collection

For deep sequencing, Basecalls were performed performed using CASAVA.
For FACS, data were collected by using the software DIVA 8.01.

Data analysis

For FACS analysis, the software DIVA 8.01 was used.

For sequencing analyses, codes were available at https://doi.org/10.6084/m9.figshare.7411835.v5

For ChIP-seq, Single-end reads of 50bp were mapped to combined genome of mm10(Gencode GRCm38.p5) and dm6(UCSC BDGP6) by BWA (version 0.7.12-r1039, default parameter), duplicated reads were then marked with biobambam2 (v2.0.87) and only non-duplicated reads have been kept by samtools (parameter "-q 1 -F 1024" version 1.2). We followed ENCODE guideline for quality control of our data. For peak calling of H3K4me3, we used MACS2(version 2.1.1.20160309). To assure the replicability, we first finalized reproducible peaks for each group as only retained a peak if it called with stringent cutoff(macs2 -q 0.05) in one sample and at least called with lower cutoff (macs2 -q 0.5) in the other sample.

For Cut-and-Run, Paired-end reads of 50bp were mapped to combined genome of mm10(Gencode GRCm38.p5) and dm6(UCSC BDGP6) by BWA (version 0.7.12-r1039, default parameter), duplicated reads were then marked with biobambam2 (v2.0.87) and only non-duplicated properly paired reads have been kept by samtools (parameter "-q 1 -F 1804" version 1.2). To generate the bigwig tracks, we count the reads number mapped for dm6 and normalized tracks to 10 million dm6 reads(i.e. double the height if the sample have 5 million dm6 reads). We then called peaks with MACS2(version 2.1.1.20160309) in paired-end mode. finalized reproducible peaks for each group as only retained a peak if it called with stringent cutoff(macs2 -q 0.05) in one sample and at least called with lower cutoff(macs2 -q 0.5) in the other sample. Then we counted reads number for each sample at these merged reproducible peaks and Voom was used to test significant differences between WT and YBX1 KO after normalization to spike-in reads number. deepTools was used to plot the heatmap.

RNA-Seq analysis
Paired-end 100-cycle sequencing was performed on HiSeq 2000 or HiSeq 4000 sequencers, per the manufacturer's directions (Illumina). RNA-seq was mapped as previously described and HTSeq (version 0.6.1p1) was used to estimate counts per million (CPM) based on GENCODE(v24). After normalization by trimmed mean of M-values (TMM), Voom was used to identify differentially expressed genes. Volcano plots were generated using ProteinPaint viewer (St. Jude) at cloud.stjude.org hosted by DNANexus. For GSEA analysis, gene sets were put together with MSigDB database(C2, v5.1) and analyzed by using prerank mode (version 3.0). Gene ontology analysis was generated by Enrichr.

For manuscripts utilizing custom algorithms or software that are central to the research but not yet described in published literature, software must be made available to editors/reviewers. We strongly encourage code deposition in a community repository (e.g. GitHub). See the Nature Research guidelines for submitting code & software for further information.

## Data

Policy information about availability of data

All manuscripts must include a data availability statement. This statement should provide the following information, where applicable:

- Accession codes, unique identifiers, or web links for publicly available datasets
- A list of figures that have associated raw data
- A description of any restrictions on data availability

All sequencing data are deposited in GEO: https://www.ncbi.nlm.nih.gov/geo/query/acc.cgi?&acc=GSE137853&token=ktolmysothktvub
Mass spectrometry data were deposited in ProteomXchange, with project accession: PXD015670.

# Field-specific reporting

Please select the one below that is the best fit for your research. If you are not sure, read the appropriate sections before making your selection.

[x] Life sciences [ ] Behavioural & social sciences [ ] Ecological, evolutionary & environmental sciences

For a reference copy of the document with all sections, see nature.com/documents/nr-reporting-summary-flat.pdf

# Life sciences study design

All studies must disclose on these points even when the disclosure is negative.

| | |
|---|---|
| Sample size | The sample size were determined by a statistical power analysis using the online tool powerandsamplesize.com/Calculators. Most calculations were done with the intention to compare 2 means, with 2-sample, 1-sided assumption. |
| Data exclusions | No exclusion. |
| Replication | Except ChIP-seq and CUT&RUN-seq, all experiments were repeated at least 2 times. All attempts at replicating results were successful. Chromatin profiling were done with CUT&RUN or ChIP-seq with wild-type vs. Ybx1-KO with Drosophila melanogaster spike-in controls. We also obtaining consistent results between ChIP-seq and CUT&RUN |
| Randomization | No randomization. Experimental groups were Ybx1-KO and wild-type control cells. |
| Blinding | Data analysis was not blinded because the data collectors performed most analyses. However, seq data were analyzed without assumption of data trend. 'unsupervised.' |

# Reporting for specific materials, systems and methods

We require information from authors about some types of materials, experimental systems and methods used in many studies. Here, indicate whether each material, system or method listed is relevant to your study. If you are not sure if a list item applies to your research, read the appropriate section before selecting a response.

## Materials & experimental systems

| n/a | Involved in the study | |
|---|---|---|
| [ ] | [x] | Antibodies |
| [ ] | [x] | Eukaryotic cell lines |
| [x] | [ ] | Palaeontology |
| [ ] | [x] | Animals and other organisms |
| [x] | [ ] | Human research participants |
| [x] | [ ] | Clinical data |

## Methods

| n/a | Involved in the study | |
|---|---|---|
| [ ] | [x] | ChIP-seq |
| [ ] | [x] | Flow cytometry |
| [x] | [ ] | MRI-based neuroimaging |

## Antibodies

Antibodies used

Antibody Species Source Catalogue Number Dilution
Anti-JARID2 Rabbit Novus Biologicals NB100-2214 IP (4µg)
Anti-JARID2 Sheep R&D Systems AF6090 IP (4µg)
Anti-JARID2 Rabbit EMD Millipore ABE425 IP (4µg)
Anti-Ybx1 Rabbit Bethyl Laboratories A303-230A IP (4µg) WB (1:1000) Cut-Run (0.4µg)
Anti-Ybx1 Rabbit Abcam ab12148 IP (4µg) WB (1:1000)
Anti-Ybx1 Rabbit Sigma Y0396 IF (1:100)
Anti-Suz12 Rabbit Cell Signaling Technology 3737 WB (1:1000)
Anti-Ezh2 Rabbit Cell Signaling Technology 4905 WB (1:1000)
Anti-Ezh2 (clone AC22) Mouse Active Motif 39875 Cut-Run (0.4µg)
Anti-Eed Mouse ThermoFisher MA5-16314 WB (1:1000)
Anti-Eed Rabbit Invitrogen PA5-34420 WB (1:1000)
Anti-RbBP5 Rabbit Bethyl Laboratories A300-109A WB (1:1000)
Anti-G9a Rabbit Cell Signaling Technology AF1979 WB (1:1000)
Anti-SOX2 Goat Santa Cruz Sc-17319 IF (1:200)
Anti-GAPDH Mouse EMD Millipore MAB374 WB (1:2000)
Anti-beta actin Mouse Sigma Aldrich A1978 WB (1:2000)
Anti-COX IV Rabbit Cell Signaling Technology 4844 WB (1:1000)
Anti-histone H3 Rabbit Active Motif 61475 WB (1:1000)
Anti-SOX2 PE (clone 14A6A34) Mouse BioLegend 656104 FACS (1:250)
Anti-BrdU Mouse BioLegend 364106 IF (1:100)
Anti-BrdU APC (clone Bu20a) Mouse BioLegend 339808 FACS (1:500)
Anti-Phospho-S10-Histone H3-Alexa 488 (D2C8) Mouse Cell Signaling Technology 3465 FACS (1:1000)
Anti-FoxG1 (EPR18987) Rabbit Abcam Ab196868 IF (1:100)
Anti-Gbx2 Rabbit ProteinTech 21639-1-AP IF (1:100)
Anti-GFAP Rabbit Cell Signaling Technologies 12389S IF (1:100)
Anti-GFAP Mouse MilliporeSigma 1G3893 IF (1:250)
Anti-beta Tubulin Mouse Sigma Aldrich T8660 IF (1:200)
Anti-Histone H3K27me3 Rabbit Active Motif 39155 ChIP (1µg)
WB (1:1000)
Cut-Run (0.4µg)
Anti-Histone H3K4me3 Rabbit Diagenode C15410003 ChIP (1ug)
WB (1:1000)
Cut-Run (0.4ug)
Drosophila spike in control anti-H2Av Rabbit Active Motif 61686 ChIP (0.5ul) or Cut-Run (0.2ul)

Validation

All antibodies were obtained from commercial vendors. The antibodies were purchased based on previous validation from publications from highly reputable labs/sources/ENCODE project. Below we described individual validation effort:
Anti-JARID2 Rabbit Novus Biologicals NB100-2214 - validation by comparing IP and WB in wild-type and Jarid2-knockdown cells.
Anti-JARID2 Sheep R&D Systems AF6090 IP - validation by comparing IP and WB in wild-type and Jarid2-knockdown cells.
Anti-JARID2 Rabbit EMD Millipore ABE425 - validation by comparing IP and WB in wild-type and Jarid2-knockdown cells.
Anti-Ybx1 Rabbit Bethyl Laboratories A303-230A - validation by comparing IP and WB in wild-type and Ybx1-KO cells.
Anti-Ybx1 Rabbit Abcam ab12148 - validation by comparing IP and WB in wild-type and Ybx1-KO cells.
Anti-Ybx1 Rabbit Sigma Y0396 - validation by comparing IP and WB in wild-type and Ybx1-KO cells.
Anti-Suz12 Rabbit Cell Signaling Technology - Company validated it with recombinant proteins WB.
Anti-Ezh2 Rabbit Cell Signaling Technology 4905 - Company validated it with recombinant proteins WB.
Anti-Ezh2 (clone AC22) Mouse Active Motif 39875 - Company validated it with recombinant proteins WB.
Anti-Eed Mouse ThermoFisher MA5-16314 - Company validated it with recombinant proteins WB.
Anti-Eed Rabbit Invitrogen PA5-34420 - Company validated it with recombinant proteins WB.
Anti-RbBP5 Rabbit Bethyl Laboratories A300-109A - Company validated it with recombinant proteins WB.
Anti-G9a Rabbit Cell Signaling Technology AF1979 - Company validated it with recombinant proteins WB.
Anti-SOX2 Goat Santa Cruz Sc-17319 - Company validated it with recombinant proteins WB.
Anti-GAPDH Mouse EMD Millipore MAB374 - Company validated it with recombinant proteins WB.
Anti-beta actin Mouse Sigma Aldrich A1978 - Company validated it with recombinant proteins WB.
Anti-COX IV Rabbit Cell Signaling Technology 4844 - Company validated it with recombinant proteins WB.
Anti-histone H3 Rabbit Active Motif 61475 - Company validated it with recombinant proteins WB.
Anti-SOX2 PE (clone 14A6A34) Mouse BioLegend 656104 - Company validated it with recombinant proteins WB.
Anti-BrdU Mouse BioLegend 364106 - Company validated it with recombinant proteins WB.
Anti-BrdU APC (clone Bu20a) Mouse BioLegend 339808 - Company validated it with recombinant proteins WB.
Anti-Phospho-S10-Histone H3-Alexa 488 (D2C8) Mouse Cell Signaling Technology 3465 - Company validated it with recombinant proteins WB.

Anti-FoxG1 (EPR18987) Rabbit Abcam Ab196868 - Company validated it with recombinant proteins WB.
Anti-Gbx2 Rabbit ProteinTech 21639-1-AP - Company validated it with recombinant proteins WB.
Anti-GFAP Rabbit Cell Signaling Technologies 12389S - Company validated it with recombinant proteins WB.
Anti-GFAP Mouse MilliporeSigma 1G3893 - Company validated it with recombinant proteins WB.
Anti-beta Tubulin Mouse Sigma Aldrich T8660 - Company validated it with recombinant proteins WB.
Anti-Histone H3K27me3 Rabbit Active Motif 39155 - Company validated it with peptide array and ChIP. We compared ChIP and CUT&RUN by sequencing analysis.
Anti-Histone H3K4me3 Rabbit Diagenode C15410003 - Company validated it with peptide array and ChIP. We compared ChIP and CUT&RUN by sequencing analysis.
Drosophila spike in control anti-H2Av Rabbit Active Motif 61686 - Company validated it with peptide array and ChIP. We compared ChIP and CUT&RUN by sequencing analysis.

# Eukaryotic cell lines

Policy information about cell lines

**Cell line source(s)**: Human embryonic stem cells H9 were purchased from WiCell, catalog WA09. 293T cells were purchased from ATCC, catalog ATCC CRL3216. NE4C cells were purchased from ATCC, catalog ATCC CRL-2925.

**Authentication**: H9 cells were authenticated by WiCell before sending to us. We used whole genome sequencing and western blotting to authenticate genetic disruption introduced to make sublines that contained mutations. 293T and NE4C cells were authenticated by ATCC before sent to us .We used H9 and NE4C cells to generate nuclear extract for immunoprecipitation followed by western blotting or mass spectrometry. We used virus generation from the 293T cells. 293T, H9, and Ne4C cells were not used for functional analyses.

**Mycoplasma contamination**: Cells were negative for mycoplasma. Cells were checked by PCR every 3-4 months.

**Commonly misidentified lines** (See ICLAC register): None.

# Animals and other organisms

Policy information about studies involving animals; ARRIVE guidelines recommended for reporting animal research

**Laboratory animals**:
Nestin-Cre: B6.Cg-Tg(Nes-Cre)1Kln/J, described in Tronche et.al.
Eed-flox: B6;129S1-Eedtm1Sho/J, described in Yu et.al.
Ybx1: Ybx1tm1Ley/Ybx1tm1Ley, described in Lu et.al.
Sox2-eGFP: B6;129S1-Sox2tm1Hoch/J, described in Arnold et.al.

**Wild animals**: None.

**Field-collected samples**: None.

**Ethics oversight**: IACUC at St. Jude oversaw the culturing and use of the mouse animals in this study.

Note that full information on the approval of the study protocol must also be provided in the manuscript.

# ChIP-seq

## Data deposition

☒ Confirm that both raw and final processed data have been deposited in a public database such as GEO.

☒ Confirm that you have deposited or provided access to graph files (e.g. BED files) for the called peaks.

**Data access links**
*May remain private before publication.*
https://www.ncbi.nlm.nih.gov/geo/query/acc.cgi?&acc=GSE137853&token=ktolmysothktvub

**Files in database submission**:
H3K27me3-AB3-YBX1WT ChIP-seq replicate 1
H3K27me3-AB3-YBX1KO ChIP-seq replicate 1
H3K4me3-AB2-YBX1WT ChIP-seq replicate 1
H3K4me3-AB2-YBX1WT ChIP-seq replicate 2
H3K4me3-AB2-YBX1KO ChIP-seq replicate 1
H3K4me3-AB2-YBX1KO ChIP-seq replicate 2
INPUT-YBX1WT
INPUT-YBX1KO
Cut-and-Run-YBX1-WT
Cut-and-Run-YBX1-KO
RNA-YBX1-WT-116-1
RNA-YBX1-WT-116-3

RNA-YBX1-WT-93-1
RNA-YBX1-null-175-1
RNA-YBX1-null-175-6
RNA-YBX1-null
Cut-and-Run-Ezh2-YBX1WT
Cut-and-Run-Ezh2-YBX1KO
Spike-in Cut-and-Run-H3K27me3-YBX1WT replicate 1
Spike-in Cut-and-Run-H3K27me3-YBX1WT replicate 2
Spike-in Cut-and-Run-H3K27me3-YBX1KO replicate 1
Spike-in Cut-and-Run-H3K27me3-YBX1KO replicate 2

Genome browser session (e.g. UCSC)

none.

## Methodology

Replicates

H3K27me3 were analyzed by 2 replicates of CUT&RUN.
H3K4me3 were analyzed by 2 replicates
Ybx1 and Ezh2 have 1 replicate of WT and Ybx1-KO each

Sequencing depth

Almost every CHIPSEQ sample have 20M unique mapped reads, only few have less but more than 15M unique mapped reads. CUT&RUN all have more than 5M as recommended

Antibodies

Anti-Ezh2 (clone AC22) Active Motif 39875
Anti-Histone H3K27me3 Active Motif 39155
Anti-Histone H3K4me3 Diagenode C15410003
Anti-Ybx1  Bethyl Laboratories A303-230A

Peak calling parameters

For CHIPSEQ, Following guideline of ENCODE, we first use SPP(v1.1) to estimate the fragment size, and then used MACS2 (version 2.0.9 20111102) to call the peaks with "--nomodel --extsize " to the estimated fragment size by SPP with broad peak model at FDR 5%. Peaks within 100 bp were then merged by bedtools (version 2.17.0).
For CUT&RUN we called by MACS2 by default parameters using paired-end mode. We also tried SEACR but it called too many weak peaks due to high depth of our data, so we only use SEACR peaks for further filtering. As is we exclude MACS2 called peaks not overlap SEACR called peaks.

Data quality

We confirmed quality control results were good following the ENCODE guideline for CHIPSEQ, reads were then extend to the estimated fragment size and converted to bigwig files.  With clear peaks observed by inspection on Integrated Genomics Viewer (Broad Institute) and there was high consistency among replicates. We further extend reads to the estimated fragment size and counted at each called peaks, pearson correlation coefficients were calculated among replicates suggested high reproducibility.

Software

Briefly, for ChIP-seq, Single-end reads of 50bp were mapped to combined genome of mm10(Gencode GRCm38.p5) and dm6 (UCSC BDGP6) by BWA (version 0.7.12-r1039, default parameter), duplicated reads were then marked with biobambam2 (v2.0.87) and only non-duplicated reads have been kept by samtools (parameter “-q 1 -F 1024” version 1.2). We followed ENCODE guideline for quality control of our data. For peak calling of H3K4me3, we used MACS2(version 2.1.1.20160309). To assure the replicability, we first finalized reproducible peaks for each group as only retained a peak if it called with stringent cutoff(macs2 -q 0.05) in one sample and at least called with lower cutoff(macs2 -q 0.5) in the other sample.

For Cut-and-Run, Paired-end reads of 50bp were mapped to combined genome of mm10(Gencode GRCm38.p5) and dm6 (UCSC BDGP6) by BWA (version 0.7.12-r1039, default parameter), duplicated reads were then marked with biobambam2 (v2.0.87) and only non-duplicated properly paired reads have been kept by samtools (parameter “-q 1 -F 1804” version 1.2). To generate the bigwig tracks, we count the reads number mapped for dm6 and normalized tracks to 10 million dm6 reads (i.e. double the height if the sample have 5 million dm6 reads). We then called peaks with MACS2(version 2.1.1.20160309) in paired-end mode. finalized reproducible peaks for each group as only retained a peak if it called with stringent cutoff(macs2 -q 0.05) in one sample and at least called with lower cutoff(macs2 -q 0.5) in the other sample. Then we counted reads number for each sample at these merged reproducible peaks and Voom was used to test significant differences between WT and YBX1 KO after normalization to spike-in reads number. deepTools was used to plot the heatmap.

# Flow Cytometry

## Plots

Confirm that:

- [x] The axis labels state the marker and fluorochrome used (e.g. CD4-FITC).
- [x] The axis scales are clearly visible. Include numbers along axes only for bottom left plot of group (a 'group' is an analysis of identical markers).
- [x] All plots are contour plots with outliers or pseudocolor plots.
- [ ] A numerical value for number of cells or percentage (with statistics) is provided.

## Methodology

| | |
|---|---|
| Sample preparation | Cells were dissociated by accutase and counted by FITC-labeled phosphorylated serine 10 in histone H3 and APC-labeled BrdU. mouse NPCs were isolated by NeuroFluor CDr3 (STEMCELL Technologies, catalog 01800). |
| Instrument | Becton Dickinson FACSAria™ Fusion model 656700 |
| Software | DIVA 8.01 |
| Cell population abundance | About 1-3 million cells were used in each sort. Fractions ranged from 0.2% to 59% of populations. |
| Gating strategy | Primary gates are the 'live cells' and the 'single cells' gates. Positive signal gate is set to distinguish negative from positive events. Dim signals are eliminated from the gate to ensure positives. Events from the gate are back-gated to the primary gates to ensure events are well within the bounds of the initial gates. |

☒ Tick this box to confirm that a figure exemplifying the gating strategy is provided in the Supplementary Information.
